# Genetic nurture effects in depressive and anxiety disorders and symptoms, and in related traits

**DOI:** 10.1038/s41380-025-03265-w

**Published:** 2025-09-19

**Authors:** Victória Trindade Pons, Albertine J. Oldehinkel, Hanna M. van Loo

**Affiliations:** Department of Psychiatry, University of Groningen, University Medical Center Groningen, Groningen, The Netherlands

**Keywords:** Genetics, Molecular biology

## Abstract

There is an elevated risk of depression and anxiety in offspring of parents with a history of these disorders. Beyond direct transmission, parental genes may also impact offspring outcomes through the environment, in a “genetic nurture” pathway. The scarcity of relevant data has limited studies in this area, resulting in an incomplete understanding of the indirect impact of parental genes on the familial transmission of depression and anxiety. We investigated genetic nurture effects in 15,231–17,186 Dutch adults with at least one genotyped parent from Lifelines, a large general population cohort. We computed polygenic scores for transmitted (PGS-T) and non-transmitted (PGS-NT) parental haplotypes using genome-wide association studies for depression. Using mixed-effect regression models, we analyzed PGS-T and PGS-NT associations with offspring outcomes, ranging from narrow (depressive and anxiety disorders according to diagnostic criteria) to broader definitions (depressive and anxiety symptoms, neuroticism, and negative affect), measured at multiple assessment waves. Our results demonstrate a pattern of significant associations between PGS-T and offspring outcomes, consistent with direct genetic transmission (OR = 1.2–1.5; β = 0.09–0.20, p < 0.001). PGS-NT effects were approaching null across all outcomes, with some exceptions in specific assessment waves. The lack of robust associations for PGS-NT across outcomes suggests a minimal role of genetic nurture in depressive and anxiety disorders, symptoms, and related traits through parental genetic liability for depression. Though the possibility of indirect genetic effects through other genetic risk factors remains, our findings point to the genetic transmission of depression and anxiety primarily occurring via direct inheritance.

## Introduction

Depression and anxiety run in families partly due to the transmission of genetic risk variants from parents to offspring, leading to a moderate heritability of approximately 30–40% [1]. Concurrently, there is evidence supporting the influence of the familial environment, even later in life [2]. Although genetic and environmental factors are distinct, they may not operate independently in transmitting liability for depression and anxiety. A potential mechanism of gene-environment interplay in depression and anxiety involves the influence of one individual’s genes on the traits of another individual through environmental pathways. One pathway for such effects is genetic nurture, which implies an indirect genetic pathway through which heritable parental traits may influence the risk of depression and anxiety in their offspring [3].

Genetic nurture effects have been consistently reported in educational outcomes [4] and may impact other behavioral outcomes. Determining the relevance of this indirect pathway can deepen our understanding of the intergenerational transmission of depressive and anxiety disorders, which is valuable for informing family-based interventions, particularly given the substantial burden of these disorders on individuals and society as a whole [5, 6]. Moreover, genetic nurture could bias estimates of direct genetic effects, thus if present should be taken into account [7].

Research on genetic nurture effects in depression and anxiety is scarce and inconclusive. The dearth of studies on this topic can be attributed to the limited number of genotyped families with relevant phenotype data, as well as limited prediction power of current polygenic scores (PGS). Most studies investigating genetic nurture in depression and anxiety were conducted in a large sample encompassing children aged 8 and yielded mixed results across variance partitioning methods [8–10]. Only one study has examined genetic nurture on adult depression, using the UKBiobank data. This study provided preliminary evidence for genetic nurture through associations of parental PGS for broad depression and cannabis use disorder to offspring neuroticism and broad depression, respectively, over and above the effects of the offspring’s own PGS [11]. Additionally, another study suggested a role for genetic nurture through a significant shrinkage (30–50%) in genome-wide association study (GWAS) estimates of depressive symptoms when comparing population to within-family estimates [12].

The present study examined genetic nurture effects through genetic liability for depression in 15,231–17,186 parent-offspring trios and pairs from Lifelines, a large biobank from the Netherlands. We constructed PGSs based on transmitted (PGS-T) and non-transmitted (PGS-NT) parental haplotypes [13]. The PGS-NT can act as a proxy for genetic nurture: a significant association with offspring outcomes can only be indirect, mediated by the environment. Due to the random segregation of alleles, both the PGS-T and PGS-NT contribute equally to genetic nurture; however, the PGS-NT reflects this unconfounded by direct genetic effects, which are also captured by the PGS-T. Unlike previous studies that focused on one or two broad definitions, we analyzed a range of outcomes related to depression and anxiety in the offspring, from narrow (disorders measured with diagnostic criteria) to broader measures (symptoms, neuroticism, and negative affect), assessed in multiple waves.

## Methods and materials

### Sample and procedure

We analyzed data from Lifelines, a multi-disciplinary prospective population-based three-generation cohort study examining the health and health-related behaviors of 167,729 persons living in the North of the Netherlands. Lifelines employs a broad range of procedures to assess biomedical, socio-demographic, behavioral, physical, and psychological factors that contribute to the health and disease of the general population, with a special focus on multi-morbidity and complex genetics. Data collection was accomplished at three general assessments, along with additional assessments. The baseline assessment took place between 2007 and 2013, followed by a second wave between 2014 and 2017, and a third wave between 2019 and 2023. The cohort profile and assessments are described in detail elsewhere [14]. This study included genotyped offspring with at least one parent also genotyped. The sample size for each analysis depended on phenotype data availability, with a maximum of 17,186 offspring comprising 2791 trios (both parents and offspring genotyped), 5252 father-offspring pairs, and 9143 mother-offspring pairs.

### Outcomes

#### Depressive and anxiety disorders

Major depressive disorder (MDD), dysthymia, generalized anxiety disorder (GAD), social phobia, agoraphobia, and panic disorder were assessed with the Mini-International Neuropsychiatric Interview (MINI) standardized diagnostic interview [15] at all three general assessments, and established based on DSM-IV criteria. Current MDD, dysthymia, GAD, social phobia, and panic disorder were defined as the presence of the required symptoms in the past 2 weeks, 2 years, 6 months, 1 month, and 1 month, respectively (American Psychiatric Association 2000). Agoraphobia was assessed without a specific duration criterion. Lifetime MDD, GAD, social phobia, and agoraphobia were assessed during an additional measurement wave with the Lifetime Depression Assessment Self-report [16], which included the same symptom criteria to determine whether participants ever met the diagnostic criteria for these disorders according to DSM-IV. A lifetime MDD measure was also collected in a version of the MINI that included lifetime items (wave 3, Supplementary Information). For the main analyses, disorder outcomes were created by aggregating all available measurements, meaning that individuals were classified as cases if they met the criteria for a disorder at least one of the assessments. Additionally, we conducted supplementary analyses using the outcomes from each assessment wave separately to examine wave-specific effects.

#### Depressive and anxiety symptoms

Symptom counts were calculated as the average of MINI symptom sum scores for MDD and GAD across all available assessments for an individual. Averaging, rather than taking the maximum, was chosen to better capture vulnerability to depression and anxiety over time while minimizing the impact of any single assessment. The sum scores of MDD symptoms ranged from 0 to 9 and the sum scores of GAD symptoms from 0 to 7 in each wave. At baseline, a smaller sample had data on every MINI item available, because then all symptoms were assessed only if at least one core item was present. We performed supplementary analyses using symptom scores from each assessment wave separately.

#### Neuroticism

Neuroticism was assessed using the Neuroticism subscale of the Revised NEO Personality Inventory [17] at baseline. The subscale covers the facets of anxiety, anger/hostility, depression, self-consciousness, impulsiveness, and vulnerability. Each facet includes 8 items, which were rated on a 5-point Likert scale. The initial questionnaire excluded the depression and anxiety facets to limit the total length of the questionnaires, but these were added in the later versions. To maximize the use of available data across all participants and maintain consistency, we computed scores excluding the depression and anxiety facets for our analyses for all participants. This 4-facet measure of neuroticism was highly correlated with the measure based on the full version (r = 0.83) and had internal reliability (Cronbach’s alpha) of 0.74. Missing items were imputed with the facet mean of an individual if at least one item was present for that facet (Supplementary Information). Additionally, we used the smaller subset of participants who had data for the complete 6-facet measure of neuroticism in sensitivity analyses.

#### Negative affect

The Positive and Negative Affect Schedule [18] was used to collect negative affect with a 10-item mood scale assessing the frequency of feeling irritable, ashamed, upset, nervous, guilty, scared, hostile, jittery, afraid, and distressed in the past four weeks. Cronbach’s alpha was 0.87. The questionnaire was administered at baseline, and items could be answered on a 5-point Likert scale, resulting in a sum score ranging from 10 to 50.

### Genotyping data

Imputed genetic data was available for 79,988 Lifelines participants genotyped across three batches. Quality control (QC) of markers and samples was performed separately per batch. The pre-imputation QC criteria are described in detail in Supplementary Information. In brief, markers with a high missing rate or low minor allele frequency, markers that were duplicated or monomorphic, and markers that deviated significantly from Hardy-Weinberg equilibrium were removed. Post QC data from each array was imputed using the Sanger Imputation Service with the Haplotype Reference Consortium v1 as reference panel. To get a common set of markers for parent and offspring genotyped in any array, we selected an overlap of imputed markers with imputation quality scores equal to or above 0.8 across arrays. Samples that had a high missing rate, heterozygosity outliers, or were identified as mix-ups were filtered out. Samples were restricted to European ancestry based on a principal component analysis using the 1000 Genomes reference to avoid confounding due to broad population stratification. In cases of individuals genotyped in more than one array, data from the most recent batch was used.

### Non-transmitted alleles inference

We used our newly developed haplotype-based approach to differentiate between transmitted and non-transmitted alleles in parent-offspring trios and pairs, which we previously validated in our study of genetic nurture effects in educational outcomes in Lifelines [13]. In short, we used SHAPEIT5 to estimate haplotypes including pedigree information [19]. Offspring haplotypes were then compared to parental haplotypes using tiles of 150 adjacent markers on each chromosome. The best match between the parent and offspring tiles, taking recombination spots into account, was used to determine which parental tiles were transmitted to the offspring. The remaining non-transmitted alleles were recorded in a separate dataset, and for parent-offspring pairs, the non-transmitted alleles of the unobserved parent were set as missing. This method was validated by comparison with standard software in parent-offspring trios and found a concordance rate for the non-transmitted alleles of 99.8%. Furthermore, the identification of non-transmitted alleles was confirmed to be unaffected by missing parental data through simulations of pairs from trios.

### Polygenic scores

We computed a PGS for depression based on the largest GWAS available at the time of analysis [20], which included individuals who reported seeking help from a physician for nerves, anxiety, tension, or depression, and formally diagnosed depression patients. The summary statistics included 170,756 cases and 329,443 controls. A total of 934,468 markers overlapping with genotype data were included in the final score. Additionally, because broad phenotyping can capture genetic signals that are not specific to depression [21], we performed supplementary analyses with PGS based on MDD assessed by structured diagnostic interviews and electronic health records, which included 44,591 cases and 97,674 controls [22]. The PGS for MDD included 1,131,512 markers that overlapped with the genotype data.

SNP effects were re-weighted using the ‘auto’ setting from LDpred2 [23], a Bayesian method that leverages trait-specific genetic architecture and linkage disequilibrium (LD) data from a chosen reference panel. Variants included in the PGS were restricted to an extended set of HapMap3 variants and the provided UK Biobank reference LD panel [24]. For each offspring, PGSs were created based on transmitted and non-transmitted datasets. PGS-T and PGS-NT were defined as the sum of the PGS based on paternal and maternal transmitted and non-transmitted haplotypes, respectively. The value of the missing PGS-NT in parent-offspring pairs was imputed with the average PGS of the observed parents and standardized based on the full PGS-T (Supplementary Information).

### Statistical analyses

Statistical analyses were performed in R version 4.2.1 [25]. We fitted mixed-effects regression models to estimate the associations between the PGS-T and PGS-NT with the outcomes. Offspring sibling relationships were accounted for by including a random effect of family ID on the intercept. We used mixed-effects logistic regression for binary outcomes using the package ‘*GLMMadaptive*’ [26] and mixed-effects linear regression for continuous outcomes with the ‘*lme4*’ package [27] and *p*-values calculated with ‘*lmerTest*’ [28]. Age at first assessment, sex, and genotyping array were included as covariates in all models. To account for population stratification and batch effects, PGSs were regressed on the first ten principal components, and the residual variances following this step were used in the models. We fitted a separate model for each outcome. Dichotomous outcomes were coded as 0 (control) or 1 (case). Age at first assessment, neuroticism, and negative affect were standardized to have a mean of 0 and variance of 1. To assess assortative mating in the parental generation of our sample, we computed the Pearson correlation between maternal and paternal broad depression PGSs. High assortative mating for a trait can confound genetic nurture effects detected with a PGS-NT.

## Results

### Sample descriptives

Sample sizes and prevalence or mean values for each outcome are presented in Table 1. The majority of the offspring were female (61.9%), with an average age of 30.7 years at the first assessment. The distributions of outcomes and PGS were mostly comparable between parent-offspring trios and pairs, although offspring in pairs showed slightly higher prevalences for the disorders and were at the higher tail for broad depression PGS compared to those in trios.

**Table 1:** Sample descriptives for all offspring and split by parent-offspring trios and pairs.

	All		Trios		Pairs	
	N	Prevalence or mean (SD)	N	Prevalence or mean (SD)	N	Prevalence or mean (SD)
**Offspring age at assessment**	17186	30.79 (8.77)	2791	29.38 (8.20)	14395	31.06 (8.85)
**Sex (female)**	17186	61.90%	2791	59.40%	14395	62.30%
**MDD**	17186	18.52%	2791	17.91%	14395	18.63%
**Dysthymia**	16936	2.56%	2736	2.19%	14201	2.64%
**GAD**	17185	11.45%	2790	11.21%	14396	11.49%
**Panic disorder**	17113	3.34%	2770	3.57%	14344	3.35%
**Agoraphobia**	17185	7.10%	2791	6.66%	14397	7.17%
**Social phobia**	17185	4.05%	2791	3.51%	14395	4.15%
**MDD symptoms (range 0**–**9)**	15242	0.68 (1.21)	2446	0.69 (1.24)	12796	0.67 (1.21)
**GAD symptoms (range 0**–**7)**	15231	1.42 (1.80)	2447	1.25 (2.00)	12784	1.41 (1.79)
**Neuroticism (range 32**–**154)**	15849	80.20 (12.77)	2450	80.21 (12.83)	13399	80.19 (12.76)
**Negative affect (range 17**–**50)**	15654	20.78 (5.23)	2425	20.88 (5.16)	13229	20.76 (5.24)
**Offspring broad depression PGS**	17186	0.016 (0.30)	2791	0.002 (0.29)	14395	0.018 (0.30)

Prevalences are shown for categorical variables and the mean with standard deviation (SD) for continuous variables.

*MDD* major depressive disorder, *GAD* generalized anxiety disorder.

### Transmitted and non-transmitted genetic effects

**Fig. 1:**
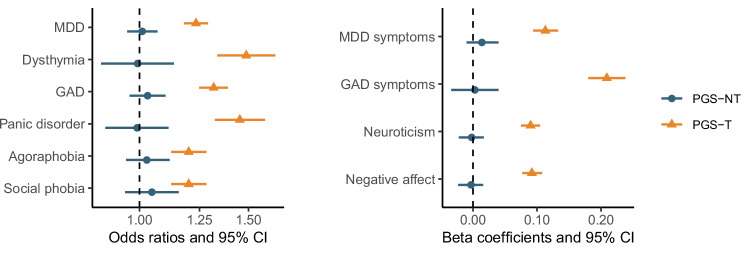
Effect estimates of the transmitted (PGS-T) and non-transmitted (PGS-NT) polygenic scores for broad depression in offspring depression and anxiety outcomes. Error bars depict 95% confidence intervals. Odds ratios (OR) and standard errors (SE) from mixed-effects logistic regression models are shown for binary outcomes, with the null effect represented by 1. Standardized betas and standard deviations (SD) from mixed-effects linear regression models are shown for continuous outcomes, with the null effect represented by 0. Offspring age at first assessment, sex, and genotyping array were included as covariates in all models. MDD major depressive disorder, GAD generalized anxiety disorder.

We found significant associations between broad depression PGS-T and all outcomes, with odds ratios ranging from approximately 1.2 to 1.5 for binary and beta coefficients ranging from 0.09 to 0.20 for continuous outcomes (Fig. 1, Table S1). PGS-NT effects were non-significant and close to 0 for all outcomes in the main analyses. A similar pattern emerged in the analyses when using the PGS for MDD, although with overall smaller effect sizes (Fig. S2). When analyzing outcomes per wave in smaller subsamples (Tables S3 and S4), we observed a few significant effects of PGS-NT for broad depression. Specifically, we found marginally significant associations of PGS-NT with GAD at wave 3 (p = 0.03), social phobia at wave 2 (p = 0.02), and GAD symptoms at wave 3 (p = 0.03). The most significant effects of PGS-NT were found in depressive symptoms at waves 1 and 3 (p < 0.01). However, these effects were not robust as they did not survive multiple testing correction using the false discovery rate (FDR) method for 31 *p*-values and lacked consistency across waves.

The low correlation (r = 0.02) between maternal and paternal broad depression PGSs suggests limited assortative mating at the polygenic score level. However, as PGSs capture only a portion of trait heritability, this likely underestimates true genetic similarity between partners due to assortative mating [29].

### Parent-of-origin analyses

**Fig. 2:**
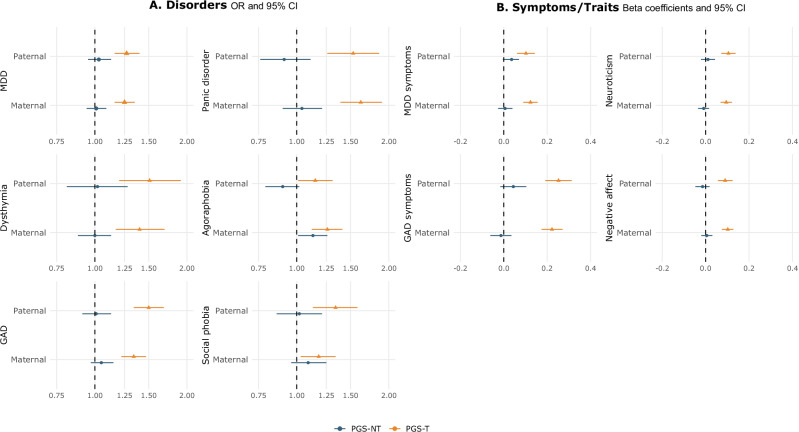
Resulting estimates of parent-of origin analyses using transmitted (PGS-T) and non-transmitted (PGS-NT) polygenic scores for broad depression split by paternal and maternal haplotypes. Error bars depict 95% confidence intervals. Odds ratios (OR) and standard errors (SE) from mixed-effects logistic regression models are shown for binary outcomes (Disorders, **A**), with the null effect represented by 1. Standardized betas and standard deviations (SD) from mixed-effects linear regression models are shown for continuous outcomes (Symptoms/Traits, **B**), with the null effect represented by 0. Offspring age at first assessment, sex, and genotyping array were included as covariates in all models. Sample sizes ranged from N = 10,508–11,934 for maternal models and from N = 7909–8043 for paternal models. MDD major depressive disorder, GAD generalized anxiety disorder.

Analyses with PGSs split by parent-of-origin resulted in an overall equal contribution from fathers and mothers in transmitted and non-transmitted genetic effects in depression and anxiety outcomes (Fig. 2). An exception arose with agoraphobia, for which we observed a positive effect of maternal PGS-NT and a negative effect of paternal PGS-NT. However, neither maternal nor paternal PGS-NT were significantly associated with agoraphobia, and since this pattern did not appear in any other anxiety disorder, we consider it a likely chance finding rather than a meaningful result. As expected based on the main analyses, the paternal and maternal transmitted PGSs were significantly associated with the outcomes.

## Discussion

### Main findings

We examined the presence of genetic nurture effects in depressive and anxiety disorders, symptoms, and related traits in a large sample of adult offspring. The results yielded robust evidence of direct genetic effects, as the transmitted broad depression PGS was significantly associated with all outcomes. We found no evidence for robust genetic nurture effects through parental genetic predisposition for depression: no stable associations were detected for the non-transmitted, parental-exclusive PGS. Additionally, we observed a general pattern of similar transmitted and non-transmitted effects in analyses with PGSs split by parent-of-origin. Our results indicate that genetic nurture through genetic risk for depression is unlikely to impact adult depression and anxiety substantially.

### Comparison with previous studies in adults

Results from our main analyses reveal a consistent pattern of significant direct genetic effects and null indirect genetic effects across all outcomes, pointing to the familial transmission of depression and anxiety occurring through direct genetic inheritance. The most comparable previous study found no association between parental broad depression PGS and depression in adult offspring either [11], which aligns with our findings. However, our results contrast with theirs for neuroticism, in that they observed a substantial effect (66% of the effect of offspring’s own PGS) of parental broad depression PGS on offspring neuroticism. This finding was not replicated in our study, despite having more precise estimates and a larger PGS-T effect size. Differences in measurement of neuroticism may explain this discrepancy, as our neuroticism measure was based on a more comprehensive questionnaire. The absence of PGS-NT effects in neuroticism and negative affect in our study provides additional evidence for a minimal role of genetic nurture in depressive and anxiety disorders, given that these stable measures are well-established indicators of vulnerability to these disorders [30].

Our wave-specific analyses suggest that genetic nurture effects may not be completely absent, as in some waves we observed significant PGS-NT effects, albeit with a small impact. Specifically, the significant associations of PGS-NT with depressive symptoms in 2 out of 3 waves are consistent with a GWAS comparison study in which within-sibship estimates for depressive symptoms were reduced by up to 50% compared to estimates from unrelated individuals [12]. Although our findings point toward some influence of genetic nurture in this outcome, the effects did not survive FDR correction, and were not observed when we averaged depressive symptoms over multiple assessments, which better captures depression risk than a single measurement. If parental genes linked to depression do impact the rearing environment to affect depressive symptoms in offspring, our results imply these effects are likely small and hard to detect.

### Comparison with childhood studies

The influence of the shared environment on depression and anxiety declines through development [31]. While this may contribute to the challenge of detecting genetic nurture in adulthood, evidence from studies in children also points towards a minimal impact of this pathway. Most studies in children have been conducted in the Norwegian Mother, Father, and Child Cohort Study (MoBa), which includes a large number of trios. These studies used variance decomposition methods, focusing on depressive and anxiety symptoms in 8-year-olds, with inconsistent results. One of the studies reported that genetic nurture accounted for 14% of the variance in depressive symptoms [8], while the other studies did not detect significant effects on children’s anxiety or depressive symptoms [9, 10]. These mixed results within the MoBa studies likely stem from differences in sample selection criteria between methods, which influenced the statistical power to detect these effects. In addition, a recognized limitation of these studies is that they may be hindered by depressive symptoms being uncommon in prepubertal children. This highlights the importance of examining these effects during adolescence and adulthood. A recent study found no robust PGS-NT effects on youth emotional problems across multiple time points from ages 3 to 17 in a population-based cohort (UK Millennium Cohort Study) [32]. Overall, our findings in adults converge with past studies in children in that indirect genetic effects contribute minimally to the variation in mood and anxiety problems.

### Implications

Investigating genetic nurture offers valuable insights into the mechanisms of intergenerational transmission. Family-based genetically informed studies have shown that parental depressive symptoms can influence offspring emotional problems, even when accounting for shared genes [33–35]. If genetic nurture effects are detected in adulthood, they may reflect cumulative processes through which early-life environmental exposures shaped by parental genotypes continue to influence the risk of mood and anxiety disorders in later stages of life [36]. However, our findings suggest that while parents pass on genetic susceptibility for depression, these genes are unlikely to shape the rearing environment in ways that elevate their children’s risk. While the rearing environment plays a crucial role in shaping vulnerability to depressive and anxiety disorder, our results suggest that this environmental pathway is not associated with parents’ genetic risk for depression. Moreover, other mechanisms of gene-environment interplay are possible and may extend beyond parent-offspring relationships, as evidenced by recent findings that the genes of partners and children can influence maternal depression [37]. Insights from these studies may inform the improvement of family-based interventions.

The extent of genetic nurture on a trait is also relevant to accurately estimates of direct genetic effects. As a form of gene-environment correlation, it can bias the estimates of direct genetic effects. Alongside assortative mating and population stratification, these effects can inflate or deflate genotype-phenotype associations and, consequently, heritability estimates in studies using only unrelated individuals [7]. Achieving corrected heritability estimates will be possible when enough data for running well-powered within-family GWAS is achieved. Given that PGS-NT effects were mostly null, our results imply that genetic nurture does not largely confound current estimates.

### Strengths and limitations

Our findings must be interpreted in light of the particular strengths and limitations of this study. First, although the availability of data from multiple waves increased the probability of detecting lifetime depression and anxiety cases, we may still have missed some cases due to missing data in later waves. Nevertheless, the alignment of our MDD lifetime prevalence with the prevalence reported by the Netherlands Institute of Mental Health and Addiction [38], along with our sample’s average age being above 30 years, which is beyond the typical onset age for MDD [39], suggests that we are not missing many cases. Second, by using our recent pipeline to include parent-offspring pairs and trios, we enhanced the statistical power and generalizability of the results. As observed in our study and supported by previous research, analyses using complete trios only may be biased as they are generally healthier than pairs [13, 32, 40]. However, the missing parent haplotype in the pairs warranted an imputation of half of their PGS-NT, which results in less precise estimates and potentially underestimated effects. Third, our sample only included participants of European ancestry, which limits the generalizability of our findings to non-Europeans or to more diverse populations. Lastly, our PGS-based study design presents a methodological challenge given the limited power of current PGSs for depression. The detection of direct and indirect genetic effects is constrained by the extent to which the PGS captures additive genetic factors, which in turn depends on GWAS sample sizes. Although the broad depression PGS based on the largest available summary statistics to date [20] effectively captured direct effects in all outcomes, there are concerns about its lack of specificity due to the broad phenotyping employed in this study [21]. To match our disorder phenotypes more closely we also used a PGS based on MDD [22]. The PGS-T of this measure was stronger associated with MDD and dysthymia than with anxiety disorders indeed, but still did not capture any indirect genetic effects. Though its predictive power is still quite limited, we found the broad depression PGS preferable due to its larger sample size. As there is the possibility that a more powerful measure may detect subtle genetic nurture effects that were missed, it is worthwhile to replicate this study when the summary statistics for the latest depression GWAS become publicly available [41].

### Future studies

Past literature on genetic nurture in depression and anxiety is scarce and focuses on broader definitions. This study is the first to examine the role of genetic nurture across a wide range of internalizing outcomes in a large sample of adults from the general population, with the advantage of observing the overall pattern across multiple phenotypes. From here on, a more complete picture can be built as more family data become available, along with triangulation with other methods. While variance decomposition methods based on genomic relationship matrixes may offer a notion of the upper bound of genetic nurture effects, PGS-based methods can be used to reveal more specific pathways. Moreover, our findings are constrained to parental genes linked to depression affecting traits related to depression and anxiety in the offspring. However, children of parents with a psychiatric disorder are at higher risk to develop one themselves, but not necessarily for the same disorder [42, 43]. Perhaps this is also true for non-transmitted genetic effects, for example, paternal PGS for cannabis use disorder has been found to be associated with offspring broad depression over and above the offspring’s own PGS [11]. There is a body of evidence for indirect genetic effects via parental genes linked to externalizing traits affecting externalizing behaviors in offspring [44, 45] that seems to be mediated by parental relationship discord [46]. Cross-trait analyses can investigate if parental genes linked to externalizing behavior, such as substance abuse, create environments that affect offspring depression and anxiety as well. This notion has been previously reported in non-genetic studies [47–50].

### Conclusion

We investigated the potential influence of genetic nurture on outcomes related to depression and anxiety and found no substantial evidence of a significant impact of non-transmitted parental genotypes linked to depression in adult offspring. While we do not rule out a small influence of genetic nurture, either through the pathway examined in this study or through other other genetic risk factors, our results suggest that genes associated with depression act primarily through direct inheritance in the familial transmission of depression and anxiety. These insights advance our understanding of the interplay between genetic and environmental influences on depression and anxiety.

## Supplementary information

Supplementary information

## Data Availability

Data may be obtained from a third party and are not publicly available. Researchers can apply to use the Lifelines data used in this study. More information about how to request Lifelines data and the conditions of use can be found on their website (https://www.lifelines.nl/researcher/how-to-apply).

## Code availability

Code for the analyses of this paper is available in a Github Gist.
