# Supplementary material for: Genetic nurture effects in depressive and anxiety disorders and symptoms, and in related traits
Source: Mol Psychiatry. 2025 Sep 19;30(12):5694–700. doi: 10.1038/s41380-025-03265-w (PMC12602338; doi:10.1038/s41380-025-03265-w)
Supplement: Supplementary file 1 — Supplementary information [file 41380_2025_3265_MOESM1_ESM.docx]

**Genetic nurture effects in depressive and anxiety disorders and symptoms, and in related traits**

**Supplementary Information**

Genotyping data

Lifelines participants were genotyped using three different arrays: the Illumina CytoSNP-12v2 array, the Infinium Global Screening Array® (GSA) MultiEthnic Disease Version 1.0, and the FinnGen Thermo Fisher Axiom® custom array, in order of release. For the CytoSNP array (released in 2020), quality control (QC) involved filtering SNPs with a minor allele frequency (MAF) above 0.001, a Hardy-Weinberg equilibrium (HWE) p-value >1e-4. A call rate threshold of 0.95 was used for both markers and samples. Sample QC included principal component analysis (PCA) to detect population outliers, and removal of duplicates, individuals with high heterozygosity and ambiguous sex, resulting in 249,249 markers and 15,422 samples. The first UGLI (GSA array) release underwent a two-step QC for marker and sample missingness thresholds (from <80% to <99%), removing monomorphic markers (MAF = 0) and those with HWE p-value >1e-6. Samples with heterozygosity >4 standard deviations from the mean, duplicates, and ambiguous sex were excluded, yielding 548,029 markers and 36,339 samples. The UGLI2 release (Affymetrix array) followed a similar two-step QC for call rates (first <80%, then <99%), removing markers with HWE p-value >1e-10 and MAF <0.02, and excluding samples with heterozygosity >4 SD from the mean, duplicates, and those with sex or family discrepancies, resulting in 462,731 markers and 28,249 samples. All arrays were imputed using the Haplotype Reference Consortium (HRC) panel via the Sanger Imputation Service. Population stratification was examined using PCA with samples from the 1000 Genomes Project, retaining only individuals of European ancestry. Post-imputation, we filtered for imputation quality (INFO >0.8) and MAF >0.05 and selected high-quality markers (HapMap3+) in each array. We then selected markers that were available in all three arrays, resulting in 1,161,061 common markers. This overlap allowed for reliable haplotype comparison across parent-offspring pairs in the sample of 19,235 offspring with at least one genotyped parent, before matching with phenotype data. Detailed QC reports for each array are available on the Lifelines wiki (<http://wiki-lifelines.web.rug.nl/>).

Outcomes

|  | **General assessments** | | | **LIDAS (Lifetime)** |
| --- | --- | --- | --- | --- |
|  | Wave 1 | Wave 2 | Wave 3 |  |
| **MDD** | X | X | X* | X |
| **Dysthymia** | X | X | X |  |
| **GAD** | X | X | X | X |
| **Panic disorder **** | X | X | X | X |
| **Agoraphobia** | X | X | X | X |
| **Social phobia** | X | X | X | X |
| **MDD symptoms** | X | X | X |  |
| **GAD symptoms** | X | X | X |  |
| **Neuroticism** | X |  |  |  |
| **Negative affect** | X |  |  |  |

*** Wave 3 also included lifetime items for MDD only. For the participants that answered both to wave 3 MINI and LIDAS (Lifetime Depression Assessment Self-report), we defined them as case if they were a case in either assessment.
** Lifetime panic disorder was defined by omitting item “in the past month, did you have such attacks repeatedly (2 or more) followed by persistent concern about having another attack?”.
MDD: major depressive disorder; GAD: generalized anxiety disorder.

Neuroticism imputation

The NEO-PI-R was imputed as follows: if at least one variable was present of a facet, the facet mean of that individual was used to impute the missing values (person-facet-mean imputation). More information about the neuroticism is available in the Lifelines wiki (<https://wikilifelines.web.rug.nl/doku.php?id=personality_sum_scores_neo>).

Neuroticism sensitivity analyses

Sensitivity analyses using the complete neuroticism version including all facets (N= 5,141) yielded comparable results to the main analyses. Using either the complete or reduced version of the NEO did not alter the results, as evidenced by effect sizes consistent with main analyses for PGS-T (β= 0.09, 95% CI 0.06 – 0.12, p<0.001) and null effects for genetic nurture (PGS-NT β= 0.00, 95% CI -0.03 – 0.03, p= 0.98)

Polygenic scores (PGS) imputation and standardization

Imputation and standardization were performed similarly to the approach described in Kong and collaborators (1). For parent-offspring trios and pairs, PGS-T were computed as the sum of the transmitted maternal and paternal haplotypic PGS. PGS-NT were calculated by summing the scores from both parents. In parent-offspring pairs where data for one parent was missing, the missing PGS-NT value for that parent was imputed using the mean score of all available scores from parents of the same type (e.g., the mean paternal PGS was used if the father’s data was missing, and the mean maternal PGS if the mother’s data was missing) before summing the scores. All PGS values, including those split by parent, were standardized using the formula z = (x-μ)/σ, where x is the raw PGS value, μ is the group mean, and σ is the group standard deviation. For trios, PGS values were standardized to have a mean of 0 and a variance of 1. The PGS-NT for parent-offspring pairs and the PGSs split by parent, the standard deviation derived from the PGS-T in trios was used in the standardization so that effect sizes would be comparable.

**Figure S1.** Distribution of the unstandardized non-transmitted broad depression polygenic score (PGS-NT) in parent-offspring pairs and parent-offspring trios. The imputation approach results in a smaller variance of PGS-NT, with values more closely grouped around the mean in parent-offspring pairs. Mean/standard deviation pairs = -4.45/0.21, trios = -4.45/0.30.

**Figure S2.** Effect estimates of the transmitted (PGS-T) and non-transmitted (PGS-NT) polygenic scores for major depressive disorder (MDD) in offspring depression and anxiety outcomes. PGS for MDD computed based on PGC MDD2 (2) summary statistics. Error bars depict 95% confidence intervals. Odds ratios (OR) and standard errors (SE) from mixed-effects logistic regression models are shown for binary outcomes, with the null effect represented by 1. Standardized betas and standard deviations (SD) from mixed-effects linear regression models are shown for continuous outcomes, with the null effect represented by 0. GAD: generalized anxiety disorder.

|  | **PGS-T** | | | | **PGS-NT** | | |
| --- | --- | --- | --- | --- | --- | --- | --- |
|  | **OR (SE)** | **95% CI** | **p** | **OR (SE)** | | **95% CI** | **p** |
| **MDD** | 1.23 (0.02) | 1.18, 1.29 | < .001 | 1.01 (0.02) | | 0.96, 1.07 | 0.70 |
| **Dysthymia** | 1.48 (0.07) | 1.34, 1.66 | < .001 | 0.99 (0.07) | | 0.87, 1.14 | 0.91 |
| **GAD** | 1.31 (0.03) | 1.25, 1.39 | < .001 | 1.03 (0.03) | | 0.96, 1.10 | 0.38 |
| **Panic disorder** | 1.45 (0.06) | 1.31, 1.60 | < .001 | 0.99 (0.06) | | 0.88, 1.11 | 0.87 |
| **Agoraphobia** | 1.20 (0.04) | 1.13, 1.28 | < .001 | 1.03 (0.04) | | 0.95, 1.12 | 0.53 |
| **Social phobia** | 1.20 (0.05) | 1.13, 1.28 | < .001 | 1.05 (0.05) | | 0.95, 1.16 | 0.37 |
|  | **Beta (SD)** | **95% CI** | **p** | **Beta (SD)** | | **95% CI** | **p** |
| **MDD symptoms** | 0.11 (0.01) | 0.09, 1.13 | < .001 | 0.01 (0.01) | | -0.10, 0.04 | 0.25 |
| **GAD symptoms** | 0.20 (0.01) | 0.18, 0.24 | < .001 | 0.00 (0.02) | | -0.03, 0.04 | 0.87 |
| **Neuroticism** | 0.09 (0.01) | 0.07, 0.10 | < .001 | 0.00 (0.01) | | -0.02, 0.02 | 0.84 |
| **Negative affect** | 0.09 (0.01) | 0.07, 0.10 | < .001 | 0.00 (0.01) | | -0.02, 0.01 | 0.74 |

**Table S1.** Main results of polygenic scores based on transmitted (PGS-T) and non-transmitted (PGS-NT) parental haplotypes on offspring outcomes. Odds ratios (OR) and standard errors (SE) from mixed-effects logistic regression models are shown for binary outcomes. Standardized beta coefficients and standard deviations (SD) from mixed-effects linear regression models are shown for continuous outcomes. Offspring age at first assessment, sex and genotyping array were included as covariates in the models. MDD: major depressive disorder; GAD: generalized anxiety disorder.

|  | **Maternal** | | | | | | **Paternal** | | | | | | |
| --- | --- | --- | --- | --- | --- | --- | --- | --- | --- | --- | --- | --- | --- |
|  | **PGS-T** | | | **PGS-NT** | | | **PGS-T** | | | **PGS-NT** | | | |
|  | **OR** | **95% CI** | **p** | **OR** | **95% CI** | **p** | **OR** | **95% CI** | **p** | **OR** | **95% CI** | **p** |  |
| **MDD** | 1.25 | 1.16,1,35 | <0.001 | 1.01 | 0.94,1.09 | 0.76 | 1.27 | 1.16,1.40 | <0.001 | 1.03 | 0.95,1.13 | 0.45 |  |
| **Dysthymia** | 1.40 | 1.17,1.69 | <0.001 | 1.00 | 0.88,1.13 | 0.99 | 1.51 | 1.20,1.91 | <0.001 | 1.02 | 0.81,1.28 | 0.87 |  |
| **GAD** | 1.34 | 1.22,1.47 | <0.001 | 1.05 | 0.97,1.15 | 0.24 | 1.50 | 1.34,1.68 | <0.001 | 1.01 | 0.91,1.13 | 0.82 |  |
| **Panic disorder** | 1.62 | 1.39,1.90 | <0.001 | 1.04 | 0.90,1.21 | 0.59 | 1.53 | 1.26,1.86 | <0.001 | 0.91 | 0.76,1.11 | 0.35 |  |
| **Agoraphobia** | 1.26 | 1.12,1.41 | <0.001 | 1.13 | 1.01,1.26 | 0.03 | 1.15 | 1.01,1.31 | 0.03 | 0.90 | 0.79,1.02 | 0.11 |  |
| **Social phobia** | 1.18 | 1.03,1.34 | 0.01 | 1.09 | 0.96,1.25 | 0.17 | 1.34 | 1.13,1.58 | 0.001 | 1.02 | 0.86,1.21 | 0.83 |  |
|  | **Beta (SD)** | **95% CI** | **p** | **Beta (SD)** | **95% CI** | **p** | **Beta (SD)** | **95% CI** | **p** | **Beta (SD)** | **95% CI** | **p** |  |
| **MDD symptoms** | 0.12 (0.01) | 0.09,0.15 | <0.001 | 0.01 (0.01) | -0.02,0.04 | 0.68 | 0.10 (0.02) | 0.06,0.14 | <0.001 | 0.04 (0.02) | -0.04,0.07 | 0.08 |  |
| **GAD symptoms** | 0.22 (0.02) | 0.17,0.27 | <0.001 | -0.01 (0.02) | -0.06,0.03 | 0.59 | 0.25 (0.03) | 0.19,0.31 | <0.001 | 0.04 (0.03) | -0.01,0.10 | 0.15 |  |
| **Neuroticism** | 0.09 (0.01) | 0.07,0.12 | <0.001 | -0.01 (0.01) | -0.03,0.16 | 0.47 | 0.10 (0.02) | 0.08,0.13 | <0.001 | 0.01 (0.02) | -0.02,0.04 | 0.50 |  |
| **Negative affect** | 0.10 (0.01) | 0.07,0.13 | <0.001 | 0.05 (0.01) | -0.02,0.03 | 0.67 | 0.09 (0.02) | 0.05,0.12 | <0.001 | -0.01 (0.02) | -0.5,0.01 | 0.39 |  |

**Table S2.** Results of analyses using transmitted (PGS-T) and non-transmitted (PGS-NT) polygenic scores for broad depression split by paternal and maternal haplotypes. Odds ratios (OR) from mixed-effects logistic regression models are shown for binary outcomes. Standardized beta coefficients and standard deviations (SD) from mixed-effects linear regression models are shown for continuous outcomes. Offspring age at first assessment, sex and genotyping array were included as covariates in the models. Sample sizes ranged from N=10,508-11,934 for maternal models and from N=7,909-8,043 for paternal models. MDD: major depressive disorder, GAD: generalized anxiety disorder.

| **Outcome** |  | **Wave 1** | | | **Wave 2** | | | **Wave 3** | | | **Lifetime** | | |
| --- | --- | --- | --- | --- | --- | --- | --- | --- | --- | --- | --- | --- | --- |
|  |  | **OR (SE)** | **p** | **N** | **OR (SE)** | **p** | **N** | **OR (SE)** | **p** | **N** | **OR (SE)** | **p** | **N** |
| **MDD** | **PGS-T** | 1.43 (0.09) | < .001 | 15837 | 1.25 (0.07) | < .001 | 10647 | 1.28 (0.08) | < .001 | 8497 | 1.34 (0.04) | < .001 | 9612 |
|  | **PGS-NT** | 1.12 (0.09) | 0.18 |  | 0.96 (0.07) | 0.57 |  | 1.05 (0.08) | 0.54 |  | 0.99 (0.03) | 0.82 |  |
| **DYS** | **PGS-T** | 1.48 (0.12) | < .001 | 15538 | 1.48 (0.12) | < .001 | 10322 | 1.55 (0.16) | < .001 | 6895 | – | – | – |
|  | **PGS-NT** | 1.09 (0.11) | 0.35 |  | 0.88 (0.09) | 0.22 |  | 1.08 (0.14) | 0.51 |  |  |  |  |
| **GAD** | **PGS-T** | 1.35 (0.05) | < .001 | 15837 | 1.58 (0.06) | < .001 | 10647 | 1.31 (0.07) | < .001 | 8487 | 1.38 (0.07) | < .001 | 4438 |
|  | **PGS-NT** | 0.92 (0.05) | 0.10 |  | 0.99 (0.05) | 0.78 |  | 1.14 (0.07) | 0.03 |  | 1.11 (0.08) | 0.16 |  |
| **PD** | **PGS-T** | 1.40 (0.14) | < .001 | 15837 | 1.60 (0.22) | < .001 | 10647 | 1.39 (0.17) | 0.01 | 8484 | 1.46 (0.07) | < .001 | 15837 |
|  | **PGS-NT** | 0.97 (0.13) | 0.79 |  | 0.99 (0.17) | 0.93 |  | 0.97 (0.16) | 0.81 |  | 0.98 (0.06) | 0.75 |  |
| **AGPH** | **PGS-T** | 1.21 (0.05) | < .001 | 15837 | 1.28 (0.08) | < .001 | 10647 | 1.28 (0.09) | < .001 | 8484 | 1.22 (0.06) | <. 001 | 4435 |
|  | **PGS-NT** | 1.04 (0.06) | 0.50 |  | 1.01 (0.08) | 0.90 |  | 0.96 (0.09) | 0.66 |  | 1.05 (0.07) | 0.41 |  |
| **SPH** | **PGS-T** | 1.52 (0.12) | < .001 | 15837 | 1.23 (0.09) | < .01 | 10647 | 1.26 (0.10) | < .01 | 8491 | 1.16 (0.07) | 0.01 | 4435 |
|  | **PGS-NT** | 0.96 (0.11) | 0.67 |  | 1.25 (0.12) | 0.02 |  | 0.99 (0.10) | 0.88 |  | 1.01 (0.08) | 0.94 |  |

**Table S3.** Odds ratios (OR) and standard errors (SE) of the associations of transmitted (PGS-T) and non-transmitted (PGS-NT) polygenic scores on disorders split by assessment wave. No PGS-NT associations were significant after false discovery rate correction for 31 p-values. MDD: major depressive disorder, DYS: dysthymia, GAD: generalized anxiety disorder, SPH: social phobia, AGPH: agoraphobia, PD: panic disorder.

| **Outcome** |  | **Wave 1** | | | **Wave 2** | | | **Wave 3** | | |
| --- | --- | --- | --- | --- | --- | --- | --- | --- | --- | --- |
|  |  | **Beta (SD)** | **p** | **N** | **Beta (SD)** | **p** | **N** | **Beta (SD)** | **p** | **N** |
| **MDD symptoms** | **PGS-T** | 0.25 (0.03) | < .001 | 8214 | 0.17 (0.02) | < .001 | 10647 | 0.14 (0.02) | < .001 | 8475 |
|  | **PGS-NT** | 0.09 (0.03) | < .01 |  | 0.01 (0.02) | 0.65 |  | 0.07 (0.03) | < .01 |  |
| **GAD symptoms** | **PGS-T** | 0.23 (0.02) | < .001 | 8277 | 0.17 (0.01) | < .001 | 10646 | 0.14 (0.01) | < .001 | 8465 |
|  | **PGS-NT** | 0.03 (0.03) | 0.21 |  | -0.01 (0.02) | 0.96 |  | 0.04 (0.02) | 0.03 |  |
| **Neuroticism** | **PGS-T** | 0.09 (0.01) | < .001 | 15849 | – | – | – | – | – | – |
|  | **PGS-NT** | 0.00 (0.01) | 0.84 |  |  |  |  |  |  |  |
| **Negative affect** | **PGS-T** | 0.09 (0.01) | < .001 | 15654 | – | – | – | – | – | – |
|  | **PGS-NT** | 0.00 (0.01) | 0.74 |  |  |  |  |  |  |  |

**Table S4**. Effects sizes (beta coefficients) and standard deviations (SD) of the associations of transmitted (PGS-T) and non-transmitted (PGS-NT) polygenic scores on disorders split by available assessment. Mixed-effect linear regression was used for neuroticism and negative affect, while symptoms were analyzed using mixed-effect Poisson regression given the count data distribution of these variables. Note that this was not feasible in the main analyses due to the data aggregation which averaged the symptoms into non-whole numbers. No PGS-NT associations were significant after false discovery rate correction for 31 p-values. MDD: major depressive disorder; GAD: generalized anxiety disorder.

|  | **Wave 1**  N= 15,837 | **Wave 2**  N= 10,647 | **Wave 3**  N= 8,505 | **Lifetime (LIDAS only)**  N= 4,438 |
| --- | --- | --- | --- | --- |
| **Offspring birth year range** | 1945 – 1995 | 1946 – 1999 | 1948 – 2004 | 1948 – 2000 |
| **Offspring age** mean (SD) | 31.5 (8.6) | 35.3 (9.3) | 41.2 (10.5) | 38.2 (10.1) |
| **Parental birth year range** | 1919 – 1974 | 1920 – 1977 | 1922 – 1982 | 1922 – 1977 |
| **Parental age** mean (SD) | 59.1 (8.3) | 61.28 (7.9) | 66.5 (8.3) | 65.5 (8.1) |
| **Sample overlap**  (%) | – | 63.3% | 47.5% | 25.8% |

**Table S5.** Sample characteristics across assessment waves. The largest available sample subset per wave with relevant phenotypic data is shown. Parental age was calculated as the average of maternal and paternal ages for parents, with the available parent's age used when one was missing. Sample overlap shows the percentage of participants from Wave 1 present in subsequent waves. Participant availability varies across waves due to the design of the Lifelines cohort and additional assessments. Further details on Lifelines assessments and response rates are available elsewhere (3).

**References**

1. Kong A, Thorleifsson G, Frigge ML, Vilhjalmsson BJ, Young AI, Thorgeirsson TE, et al. The nature of nurture: Effects of parental genotypes. Science. 2018 Jan 26;359(6374):424.

2. Wray NR, Ripke S, Mattheisen M, Trzaskowski M, Byrne EM, Abdellaoui A, et al. Genome-wide association analyses identify 44 risk variants and refine the genetic architecture of major depression. Nat Genet. 2018 May;50(5):668–81.

3. Sijtsma A, Rienks J, van der Harst P, Navis G, Rosmalen JGM, Dotinga A. Cohort Profile Update: Lifelines, a three-generation cohort study and biobank. Int J Epidemiol. 2022 Oct 1;51(5):e295–302.
